# Heartbeat-evoked potentials reveal interoceptive dysfunction in clinical disorders: Experimental frameworks and promising applications

**DOI:** 10.1017/S003329172610453X

**Published:** 2026-07-13

**Authors:** Jie Wang, Enshuo Li, Xingwei An, Dong Ming

**Affiliations:** 1 Tianjin Key Laboratory of Brain Science and Neuroengineering, Medical School, Tianjin University, Tianjin 300072, PR China; 2 State Key Laboratory of Advanced Medical Materials and Devices, Tianjin University, Tianjin 300072, PR China; 3 Haihe Laboratory of Brain-Computer Interaction and Human-Machine Integration, Tianjin 300000, PR China

**Keywords:** bottom-up, heartbeat-evoked potential (HEP), interoception, predictive coding framework, perception-filtering model, top-down

## Abstract

Interoception refers to the ability to perceive and integrate physiological signals originating from within the body, such as heartbeat and respiration. This process involves both bottom-up and top-down. As a key neurophysiological marker of interoception, the heartbeat-evoked potential (HEP) reflects the cortical processing of cardiac signals in the brain. In this review, we first outline the neural mechanisms underlying interoception and HEP, followed by a comprehensive overview of the methodologies commonly employed in HEP research. Based on the directionality of interoceptive information flow, we categorize HEP-related experimental designs into three types: bottom-up bodily sensory input, top-down predictive perception, and top-down regulation. Additionally, we explore the clinical relevance of HEP in areas such as psychiatric disorders and cardiac-related conditions. Finally, we recommend expanding research on top-down predictive perception and top-down regulation in clinical contexts.

## Introduction

Interoception refers to the processing of internal body signals (Chen et al., 2021). As a key component of bodily self-awareness, it is modulated by factors such as emotional states (e.g. anxiety and depression), attentional focus (interoceptive vs. exteroceptive), and states of consciousness (e.g. wakefulness or sleep). Therefore, in psychological and neuroscientific research, it is recognized as the foundational physiological process that underlies emotion, cognition, and self-awareness (Khalsa et al., 2018; Park & Blanke, 2019).

Recent reports suggested that interoception processes are bidirectional, encompassing both bottom-up and top-down processes (Chen et al., 2021; Desmedt et al., 2025). According to the predictive coding framework, the brain is considered to generate a hierarchy of models, comparing top-down predictions with bottom-up sensory inputs (Banellis & Cruse, 2020). When predictions do not match the actual inputs, prediction errors will be generated. These errors could be used to optimize the brain’s generative models (the brain’s predictions based on sensory inputs) to improve the accuracy of prediction sensing. Therefore, Desmedt et al. defined the interoception as ‘the bottom-up and top-down processes by which an organism senses, interprets, and integrates signals from within itself and below the skin, across conscious and nonconscious levels’ (Desmedt et al., 2025). In addition, Chen et al. incorporated top-down regulatory processes – namely, responses to interoceptive input as well as to cognitive and exteroceptive factors – into the theoretical framework of interoception, as reflected in their definition: ‘Interoception includes the processes by which an organism senses, interprets, integrates, and regulates signals from within itself’ (Chen et al., 2021). Therefore, considering the organism’s response to external stimuli, the flow of interoceptive information can be divided into three distinct processes: bottom-up sensory input, top-down predictive perception, and top-down regulatory.

To comprehensively assess and quantify an individual’s interoceptive ability, Khalsa et al. categorized the structure of interoception into multiple dimensions (Khalsa et al., 2018), such as (1) Interoceptive accuracy(IAc), which refers to the objective ability to correctly detect internal bodily signals, such as the accuracy of heartbeat detection; (2) Interoceptive sensibility (ISe), which denotes the self-reported tendency or subjective disposition to attend to and focus on internal bodily sensations, usually evaluated through self-report questionnaires; (3) Interoceptive awareness (IAw), which is the meta cognitive awareness of one’s performance in behavioral tasks, measured by the correspondence between actual performance (e.g. accuracy of heartbeat detection) and the subjective confidence in one’s performance (i.e. self-estimated accuracy of heartbeat detection). Recently, another dimension has gradually attracted attention (Coll et al., 2021; Forkmann et al., 2016): objective physiological indicators representing the transmission of visceral afferent signals, such as heart rate variability (HRV) and heartbeat-evoked potentials (HEP), also known as heartbeat-evoked responses (HER) and heartbeat evoked signals (HES). In this study, we focus on research related to HEP.

HEP is considered a neurophysiological marker of interoception, representing changes in electroencephalogram (EEG) or other bioelectrical signals caused by each heartbeat. Typically, HEP is detected and analyzed by time-locking the EEG to cardiac events, reflecting the cortical processing of cardiac activity in the brain (Coll et al., 2021). The HEP latencies range from 100 to 600 ms after the R-peak (Coll et al., 2021), which is generally observed over frontocentral regions as well as in parietal regions, with dipoles located in the anterior cingulate cortex, frontal lobe, somatosensory cortex, and right insular cortex (Müller et al., 2015; Xu et al., 2025). Studies indicate that under interoceptive conditions, HEP signals are closely related to an individual’s cardiac perception ability, with higher HEP amplitudes potentially indicating enhanced sensitivity to and processing of interoceptive signals (Judah et al., 2018; Yoris et al., 2017), which is associated with an individual’s IAc, IAw, and ISe (Coll et al., 2021). Moreover, the cardiac cycle, encompassing the systolic and diastolic phases, provides a critical temporal framework for examining how phase-specific stimuli modulate HEP and influence behavioral and emotional regulation (Raimondo et al., 2017). Notably, to ensure that HEP reflects cortical processing of heartbeats rather than cardiac electrical activity (Coll et al., 2021), cardiac field artifacts (CFA) must be removed during EEG preprocessing (see Review (Park & Blanke, 2019) for methods).

Interoception, which is fundamental to maintaining homeostasis, depends on dynamic bidirectional communication between peripheral visceral signals and central neural processing systems. Disruptions in this process, whether arising from aberrant bodily signals or dysfunctional cortical processing of interoceptive information, may contribute to impaired brain–heart interaction (BHI). Such impairments may originate from altered autonomic cardiac dynamics, disrupted afferent transmission, or dysfunctional cortical integration of visceral input. The HEP provides a direct neurophysiological index of cortical processing of cardiac afferent signals. Accordingly, disturbances in BHI are expected to manifest as alterations in HEP characteristics, including altered amplitude, latency shifts, or abnormal spatial distribution. Indeed, clinical studies have reported altered HEP patterns, suggesting impairments in cardiac afferent signaling, efferent regulation, or cortical integration mechanisms (Flasbeck et al., 2020; Pang et al., 2019; Perogamvros et al., 2019; Schmitz et al., 2020; Schulz et al., 2022; Terhaar et al., 2012). Interoceptive interventions and regulation methods could become important strategies for the treatment and management of these disorders, including behavioral approaches (Lima-Araujo et al., 2022) and neural stimulation (Xu et al., 2025). Recently, some studies have monitored the effectiveness of these interventions and regulation methods through HEP, exploring the potential application of HEP as a reliable clinical biomarker (Verdonk et al., 2024). This not only aids in optimizing treatment strategies but also improves patient prognosis and provides more precise clinical intervention guidance.

Thus, HEP offers novel insights and methodologies for investigating, diagnosing, and intervening in physiological and psychological disorders. In this, we provide an overview of the theoretical foundations of interoception and HEP and further categorize existing HEP-related experimental designs based on the directional characteristics of interoceptive information processing. We then introduce the clinical applications of HEP, including its relevance to mental health disorders, cardiovascular conditions, and other related areas. Finally, we provide recommendations for future HEP research in clinical studies to enhance our understanding of the intricate mechanisms underlying the interoceptive dysfunction.

## Experimental design of HEP

To analyze the impact of different perceptual conditions on HEP, researchers have developed a variety of experimental paradigms designed to capture and analyze HEP, facilitating comprehensive analyses tailored to diverse research objectives and questions. Some of the experimental paradigms are shown in Table 1.

**Table 1.** Experimental design of HEP Table 1. long description.

Flow type	Task	Task description
Bottom-up sensory input	Resting stats (Müller et al., 2015, Pang et al., 2019, Schmitz et al., 2021)	Participants remained seated in a relaxed state (closed or opened eyes), without any external stimuli or tasks.
	Sleep processes (Perogamvros et al., 2019, Simor et al., 2021)	Various stages of sleep were monitored, tracking physiological changes throughout the sleep cycle.
	Passive sensory tasks (Maister et al., 2017)	Participants were exposed to sensory stimuli without the need to actively respond.
Top-down predictive perception	Omission or mismatch task (Banellis & Cruse, 2020, Chennu et al., 2016, Pelentritou et al., 2024, Pfeiffer & De Lucia, 2017, Raimondo et al., 2017)	A certain proportion of omissions or mismatches is present in the regular sequence.
	Local–global paradigm (Chennu et al., 2016, Raimondo et al., 2017)	A certain proportion of local or global rule violations is presented in a regular sequence to study neural responses to rule patterns.
	Repetition suppression paradigm (Gentsch et al., 2019)	Repeated stimuli are presented to investigate the reduction in neural activity, which reflects the brain’s adaptive response to familiar stimuli.
Top-down regulation	Heartbeat Counting Task (Giusti et al., 2024, Schulz et al., 2015b, Schulz et al., 2022)	Participants silently count their heartbeats.
	Heartbeat Attention Task (Kritzman et al., 2022)	Participants focus on either their heartbeat or external stimuli as cued.
	Heartbeat Tapping Task (Zaccaro et al., 2022)	Participants tap their fingers in sync with each perceived heartbeat.
	Heartbeat Synchrony Task (Salomon et al., 2016)	Participants distinguish between stimuli that are synchronous or asynchronous with their heartbeats.
	Other cognitive processing tasks (Herrera et al., 2025, Rapp et al., 2023)	Participants engage in tasks involving attention, memory or emotional evaluation and make corresponding cognitive responses.

### Purely bottom-up sensory input

The bottom-up sensory input predominantly involves interoceptive and exteroceptive signals (such as visual, auditory, and tactile sensations) passively transmitted to the brain, modulating the brain’s electrophysiological responses without engaging active cognitive processing. Within this framework, HEP represents the brain’s passive response to cardiac activity, illustrating how the brain’s perception of cardiac signals is influenced by internal sensations or external stimuli. Resting states, sleep processes (Perogamvros et al., 2019; Simor et al., 2021), and passive sensory tasks involving visual, auditory, or other external stimuli (Maister et al., 2017) exemplify purely bottom-up sensory input.

### Top-down regulation

Responses to internal sensations or external stimuli through additional cognitive tasks represent a combination of bottom-up sensory input and top-down regulation. In this process, HEP signals can reveal how the brain actively regulates its response to cardiac signals. Researchers have investigated various task-based approaches to assess individuals’ cardiac perception abilities.

**Figure 1.**
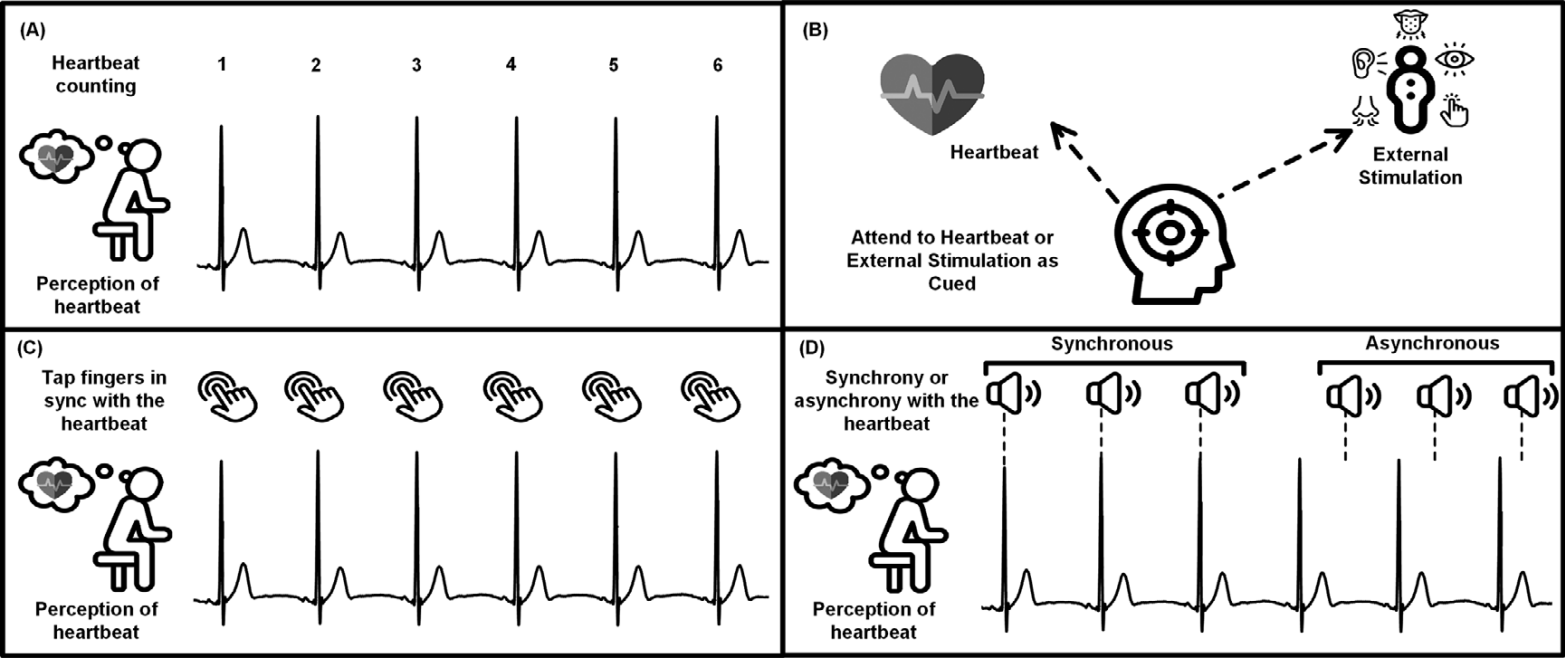
Tasks related to assessing top-down regulation of cardiac interoception. (a) Heartbeat counting task: participants silently counted their heartbeats without feeling their pulse. (b) Heartbeat attention task: participants focus either on their heartbeat or external stimuli as cued. (c) Heartbeat tapping task: participants are instructed to tap their fingers in sync with each perceived heartbeat. (d) Heartbeat synchrony task: participants distinguish stimuli that are either synchronous or asynchronous with their heartbeats. Figure 1. long description.

For example, Figure 1 illustrates representative tasks used to assess **top-down regulation of cardiac interoception**, rather than the full range of tasks related to cardiac perception. The **heartbeat counting task** requires participants to silently count their heartbeats, evaluating their focus and perception of cardiac signals (Giusti et al., 2024; Schulz et al., 2015b; Schulz et al., 2022). The **heartbeat attention task** directs participants to focus either on their heartbeat or on external stimuli as cued, thereby evaluating how interoceptive and exteroceptive attention modulate HEP (Kritzman et al., 2022). In the **heartbeat tapping task**, participants are instructed to tap their fingers in sync with each perceived heartbeat, introducing an action response component where participants externally respond to their cardiac perception (Zaccaro et al., 2022). The **heartbeat synchrony task** involves distinguishing between stimuli that are either synchronous or asynchronous with their heartbeats and assessing their capacity to integrate interoceptive and exteroceptive signals (Salomon et al., 2016).

Other more complex cognitive tasks may involve emotion induction tasks (Herrera et al., 2025; Judah et al., 2018; Rapp et al., 2023), working memory tasks (Kamp et al., 2023), multisensory stimulation, and so on.

### Top-down predictive perception

Within the predictive coding framework, top-down processes involve higher cognitive functions, such as using predictions, expectations, and prior experiences to regulate the processing of future sensory inputs (Banellis & Cruse, 2020). This perception modality is revealed through EEG measurements of the brain’s response to violations of regularity caused by unexpected stimuli or sequence interruptions, commonly referred to as mismatch negativity (MMN) (Pfeiffer & De Lucia, 2017). Similar effects are observed in the HEP (Banellis & Cruse, 2020), which reflects the brain’s anticipatory expectations of cardiac activity.

**Figure 2.**
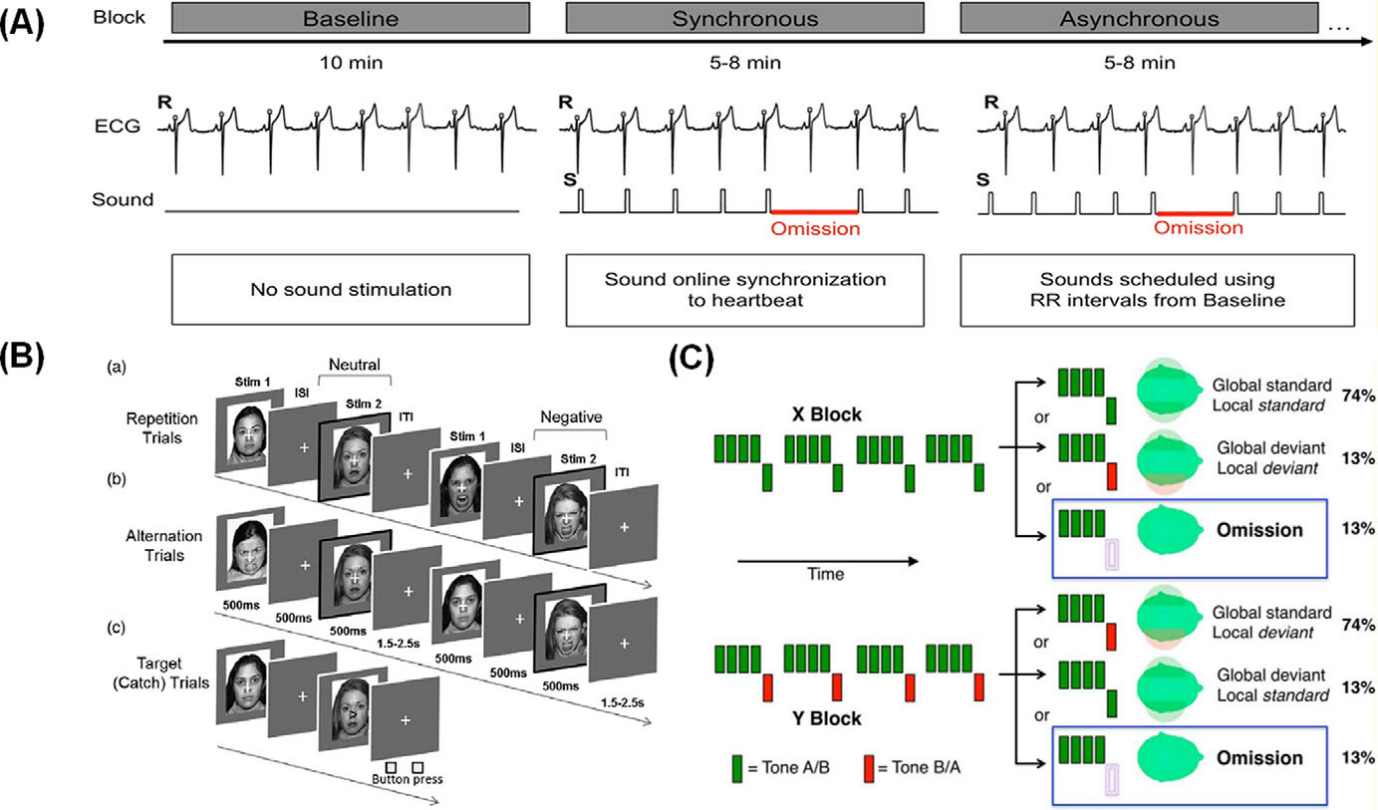
Examples of top-down predictive perception experimental design. (a) The omission in regular sequence (Pfeiffer & De Lucia, 2017). (b) The repetition suppression paradigm (Gentsch et al., 2019). (c) The local–global paradigm (Chennu et al., 2016). Figure 2. long description.

In experimental designs, researchers typically employ repetition suppression paradigms in response to frequent stimuli or investigate unexpected mismatches between predicted and presented stimuli in regular sequence paradigms to study these phenomena (Pfeiffer & De Lucia, 2017), **as shown in** Figure 2. These experimental designs are considered as an elegant way to observe pure prediction signals for expected stimuli (such as sounds) without contamination from evoked potentials (Banellis & Cruse, 2020).

**The omission** (Banellis & Cruse, 2020; Pelentritou et al., 2024; Pfeiffer & De Lucia, 2017; Raimondo et al., 2017) **or mismatch** (Raimondo et al., 2017) **in regular sequences** refers to a series of stimuli (such as auditory, visual, or other sensory inputs) arranged in a specific pattern or sequence that be presented to participants at fixed intervals. When this pattern is disrupted by omitting (omission) or replacing a predicted stimulus with a different one (mismatch) at a lower probability, prediction errors will be generated (Pfeiffer & De Lucia, 2017). Examples include the heartbeat-synchronized paradigm (Pelentritou et al., 2024; Pfeiffer & De Lucia, 2017) and the auditory local–global paradigm (Raimondo et al., 2017).

## Methods

**Figure 3.**
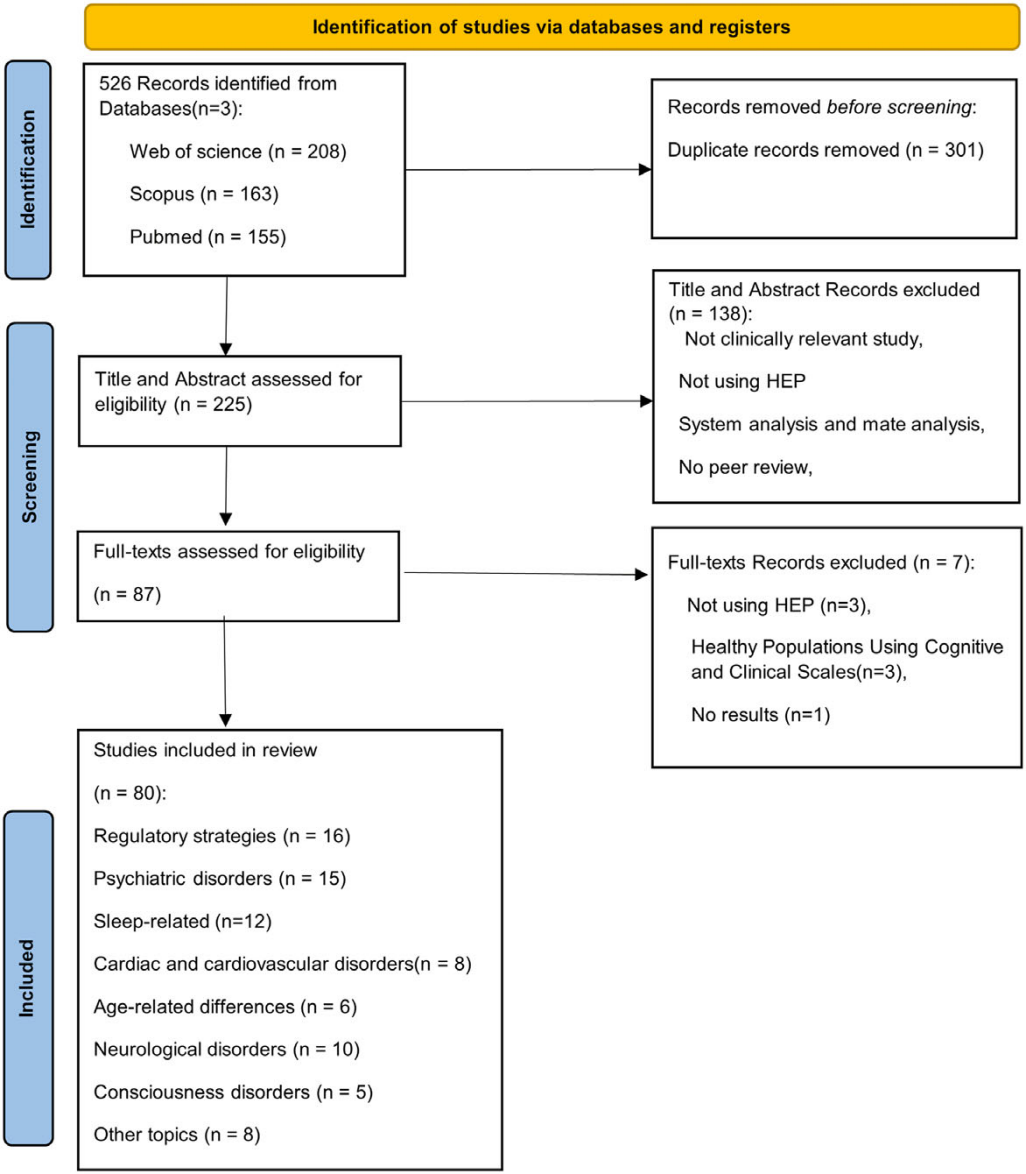
PRISMA 2020 flow chart illustrating the identification of included studies. Figure 3. long description.

To analyze the clinical applications of HEP, we conducted a systematic literature search following the PRISMA 2020 guidelines, as shown in Figure 3. Published articles were retrieved from Web of Science, Scopus, and PubMed, with the search conducted up to July 8, 2025. The search string was: (‘heartbeat evoked’ OR ‘heartbeat related’) AND (‘disorder’ OR ‘clinical’ OR ‘patient’ OR ‘diagnosis’ OR ‘treatment’ OR ‘EEG’ OR ‘MEG’ OR ‘fMRI’ OR ‘MRI’ OR ‘fNIRS’). A total of 526 article records were identified, and 301 duplicates were removed. After screening titles, abstracts, and full texts, 80 studies were included in the final review. These were categorized into eight thematic sections: regulatory strategies, psychiatric disorders, sleep, cardiac and cardiovascular disorders, age-related differences, neurological disorders, consciousness disorders, and other topics. In addition, Supplementary Tables 1 and 2 present a systematic classification of the included studies according to experimental paradigm types, along with the number of studies in each category and the commonly used task paradigms within each type.

## Results

### Regulatory strategies

In recent years, a growing body of research has integrated various neuromodulation and behavioral interventions with HEP to investigate their effects on interoceptive processing in both healthy individuals and clinical populations. For instance, meditation training has been shown to enhance attentional allocation and neural processing of cardiac signals (Gao et al., 2023; Wang et al., 2024; Yang et al., 2025), with concurrent symptom improvement in patients with posttraumatic stress disorder (PTSD) (Kang et al., 2022), reflected by increased HEP amplitude. Other body-awareness-based interventions (e.g. physical exercise, Yoris et al., 2024; and scripture recitation, Majeed et al., 2022), pharmacological agents (e.g. oxytocin, Schmitz et al., 2020, Zhou et al., 2024; peripheral epinephrine, Verdonk et al., 2024; venlafaxine, Zwienenberg et al., 2023; lipopolysaccharide, Flasbeck et al., 2025; and cortisol, Schulz et al., 2013), and neuromodulatory techniques (e.g. repetitive transcranial magnetic stimulation, Pollatos et al., 2016; Zwienenberg et al., 2023; deep brain stimulation, Xu et al., 2025; ultrasound, Strohman et al., 2024; and vagus nerve stimulation, Richter et al., 2021) have also been found to modulate interoceptive function and induce plastic changes in the spatiotemporal characteristics of HEP. Although further research is needed to confirm the reliability and clinical utility of these methods, HEP holds promise as a neurophysiological marker for evaluating the efficacy of neuromodulatory and behavioral interventions targeting interoceptive processing.

### Psychiatric disorders

Interoceptive dysfunction caused by psychiatric disorders may disrupt the BHI, leading to impairments in both passive and active signal filtering, altered perception of bodily signals, and abnormal activity in relevant brain regions.

An increasing of evidence indicates that during both passive and active cardiac-related tasks, patients with psychiatric disorders exhibit significant differences in HEP amplitude compared to healthy controls – particularly in the frontal regions (notably the right frontal cortex), central areas, and parietal lobes (Cambi et al., 2024; Flasbeck et al., 2020; Herrera et al., 2025; Judah et al., 2018; Koreki et al., 2024; Lutz et al., 2019; Müller et al., 2015; Pang et al., 2019; Terhaar et al., 2012; Yoris et al., 2017; Zhou et al., 2022). Elevated HEP amplitudes may reflect hyperprocessing of interoceptive signals. For instance, some studies have shown that increased HEP amplitude is significantly correlated with the severity of anxiety symptoms, suggesting heightened attention to internal bodily states. This intensified self-monitoring may exacerbate psychiatric symptoms and manifest as marked deficits in interoceptive processing in conditions such as PTSD (Herrera et al., 2025), obsessive-compulsive disorder (Yoris et al., 2017), and major depressive disorder (Zhou et al., 2022). Conversely, reduced processing of bodily signals may be associated with emotional blunting, diminished decision-making, and impaired cognitive functioning (Herrera et al., 2025; Müller et al., 2015; Terhaar et al., 2012).

However, some studies report no differences in HEP between individuals with patients with posttraumatic stress disorder (Schmitz et al., 2021), depersonalization/derealization disorder (Schulz et al., 2015b; Schulz et al., 2016), somatoform disorders or major depressive disorder (Schulz et al., 2022), and healthy controls, and there are still inconsistencies in the current research results on the differences in the amplitude changes of HEP between patients with depression and healthy people (Schulz et al., 2022; Terhaar et al., 2012; Zhou et al., 2022).

Additionally, increasing evidence from EEG source localization and intracranial EEG (iEEG) studies suggests that various psychiatric disorders are associated with functional abnormalities in brain regions involved in interoceptive processing. Specifically, aberrant activation or structural alterations have been observed in key interoceptive network regions such as the insula, anterior cingulate cortex (ACC), and orbitofrontal cortex (OFC) among patients with borderline personality disorder (Müller et al., 2015), anorexia nervosa (Cambi et al., 2024), and major depressive disorder (Zhou et al., 2022).

### Cardiac and cardiovascular disorders

The heart plays a crucial role in the BHI, and the incoming signals caused by its beating are transmitted to the central nervous system through sensory receptors and vagus nerve (Garfinkel & Critchley, 2016; Salamone et al., 2020) and processed and integrated into the cerebral cortex and interoceptive nervous system. Studies have demonstrated that hypertensive patients exhibit impaired perceptual abilities (Yoris et al., 2018) and significant alterations in HEP (Legaz et al., 2022). Source localization analysis revealed structural and functional abnormalities in the gray matter, insular cortex, and anterior cingulate cortex, suggesting that peripheral cardiovascular alterations may induce neurophysiological changes in central brain regions. Buot et al. have questioned the relationship between stroke volume (SV) and HEP, suggesting that HEP is not related to the activation of arterial baroreceptors, and their findings confirmed that HEP amplitudes corrected using Independent Component Analysis (ICA) showed no correlation with SV (Buot et al., 2021).

Moreover, cardiac dysfunction (Candia-Rivera & Machado, 2023b; Gray et al., 2007; Kumral et al., 2022; Limonova et al., 2024; Schulz et al., 2018) and cardiac surgery (Couto et al., 2014; Salamone et al., 2020) can disrupt the pathways underlying BHI, thereby altering the transmission and cortical processing of interoceptive signals. Such alterations offer novel insights into understanding the electrophysiological mechanisms of HEP. Patients with atrial fibrillation show a marked reduction in HEP amplitude – particularly in the right insular cortex – indicating impaired BHI and suggesting HEP as a potential electrophysiological biomarker (Kumral et al., 2022). During ventricular fibrillation, patients display attenuated or unstable HEP waveforms (Candia-Rivera & Machado, 2023b). Furthermore, HEP amplitude correlates with the severity of premature ventricular contraction symptoms (Limonova et al., 2024) and reflects cardiac proarrhythmic states (Gray et al., 2007), even predicting survival outcomes in cardiac arrest (Schulz et al., 2018). Similarly, cardiac surgeries can significantly compromise heart–brain communication, leading to reduced interoceptive sensitivity and diminished late HEP amplitudes (Couto et al., 2014, Salamone et al., 2020).

### Sleep-related

The sleep cycle mainly consists of nonrapid eye movement (NREM) sleep (categorized by depth into N1, N2, and N3, with progressively decreasing vigilance) and rapid eye movement (REM) sleep (higher vigilance, divided into phasic: with eye movements; and tonic: without eye movements). During the transition from wakefulness to deep sleep-in healthy individuals, the interoception of cardiac signals is uneven, primarily as shown by differences in HEP amplitudes across various vigilance states, with the right hemisphere showing stronger activation (Lechinger et al., 2015). Specifically, the amplitude of HEP in the frontocentral cortex decreases with increasing sleep depth (Lechinger et al., 2015) and, in particular, decreases significantly during the sleep spindle in stage N2, which may be due to spindle-associated thalamocortical suppression of visual and auditory inputs. During REM sleep, however, HEP amplitudes increase again, approaching levels seen in light sleep or wakefulness (Lechinger et al., 2015). However, some evidence suggests that early HEP components may be larger during N3 compared to N2 and REM, although no significant difference was found between N2 and REM in that study (Billeci et al., 2021). Furthermore, phasic and tonic REM show distinct modulation patterns in late HEP components (Simor et al., 2021; Simor et al., 2025), with tonic REM resembling a transitional state between wakefulness and phasic REM, indicating partial restoration of interoceptive processing during this period (Simor et al., 2021).

Sleep disorders (Seddighi & Mohebbi, 2024) such as insomnia (Wei et al., 2016), sleep deprivation (Liu et al., 2022), sleep-disordered breathing (Abolfathi & Mohebbi, 2024), and nightmare disorder (Perogamvros et al., 2019) disrupt the balance between interoception and exteroception during different sleep periods, interfering with the brain’s recovery and environmental vigilance mechanisms. Studies have shown that patients with insomnia (Wei et al., 2016) exhibit significantly enhanced late HEP components in the frontocentral regions compared to control groups, potentially involving excessive cortical processing and inhibition deficits (Wei et al., 2016). In patients with nightmares (Perogamvros et al., 2019) during REM sleep (though not replicated (Bogdány et al., 2022) and in patients with obstructive sleep apnea (Abolfathi & Mohebbi, 2024) across REM and NREM stages, HEPs show a positive shift in negative waveforms relative to healthy individuals. Moreover, HEP alterations may be reversible: in children with mild to moderate sleep-disordered breathing, abnormal HEP patterns normalize after adenotonsillectomy (Baumert et al., 2015), while 24-hour acute sleep deprivation in healthy adults does not significantly alter HEP amplitudes, suggesting that HEP disruptions are more likely linked to chronic rather than short-term sleep disturbances (Liu et al., 2022).

### Neurological disorders

Abnormal HEP patterns have been implicated in the pathophysiology of multiple neurological disorders, as structural or functional abnormalities in specific brain regions can significantly alter their spatiotemporal features. In epilepsy, characterized by hypersynchronous neuronal activity, disrupted cortical excitability and connectivity may interfere with normal heart–brain interactions. Drug-resistant epilepsy patients show stronger coupling between cardiac indices and cortical activity across all EEG frequency bands than healthy controls (Melo et al., 2022). In contrast, patients with functional seizures exhibit reduced HEP amplitude in central and right prefrontal regions during episodes, accompanied by weakened HRV–HEP coupling, suggesting impaired cortical representation of interoceptive signals (Elkommos et al., 2023; Flasbeck et al., 2024). Moreover, respiratory modulation further affects cardiac interoceptive processing in epilepsy (Stoupi et al., 2024).

HEP research based on intracranial EEG in epilepsy patients has further revealed the complex topological organization underlying heart–brain interactions. This network not only involves canonical interoceptive regions such as the bilateral thalamus, insula, amygdala, and anterior cingulate cortex but also extends to higher-order cognitive control areas, including the dorsolateral prefrontal cortex, superior parietal lobule, and superior temporal cortex (Wang et al., 2025). Notably, HEP activity within the default mode network and the right anterior insula provides direct electrophysiological evidence linking cardiac monitoring functions to the neural basis of self-awareness (Babo-Rebelo et al., 2016).

Beyond epilepsy, distinctive HEP alterations have also been reported in other neurological disorders, including dementia (Birba et al., 2022), multiple sclerosis(Salamone et al., 2018), diabetic neuropathy (Leopold & Schandry, 2001), and attention-deficit/hyperactivity disorder (Rapp et al., 2023), suggesting that HEP may serve as a sensitive biomarker reflecting both neural network instability and dysregulation of autonomic function.

### Age-related differences

Existing studies have shown that individuals’ sensitivity to internal sensory signals and their neural representations show dynamic changes at different stages of life development. Developmental research has uncovered preliminary evidence for behavioral and neural sensitivity to interoceptive signals during infancy (Maister et al., 2017). Neurophysiological evidence indicates that infants not only perceive physiological changes but also exhibit elementary integration of interoceptive information with exteroceptive stimuli. Notably, infant HEP show significant modulation during emotional processing, particularly exhibiting enhanced responses to negative emotional stimuli (Maister et al., 2017; Weijs et al., 2023). During adolescence, studies reveal a positive correlation between frontal-central HEP amplitude and IAc, suggesting HEP may serve as a neural marker for interoceptive ability development (Mai et al., 2018).

The aging process manifests distinct degradation in heart–brain interaction mechanisms. Longitudinal data demonstrate age-related decreases in temporal complexity of the HES (López Pérez et al., 2023), accompanied by characteristic alterations in frontal-central HEP amplitude (Kamp et al., 2021). Importantly, older adults show an inverse relationship between HEP amplitude and subjective cognitive assessments, while reduced HES complexity objectively predicts cognitive decline. Furthermore, cardiac rhythm exerts amplified modulatory effects on cortical perception in the elderly (Aprile et al., 2025). These findings together reveal the important relationship between the changes in the coupling mechanism of the heart and brain and the degradation of cognitive function in the elderly.

### Consciousness disorder

Disorders of consciousness provide evidence suggesting that cardiac activity may play a role in the neurobiological processes underlying consciousness (Candia-Rivera, 2022). Current research evidence suggests that the HEP can serve as a reliable neuro-electrophysiological marker for detecting residual consciousness in patients with disorders of consciousness. The integration of multidimensional HEP features (Candia-Rivera et al., 2021; Candia-Rivera & Machado, 2023a; Fló et al., 2024; Liuzzi et al., 2024) (such as time-domain characteristics, frequency-domain components, nonlinear dynamic properties, and brain network connectivity metrics) with machine learning-based classification models enables high-precision differential diagnosis between patients with unresponsive wakefulness syndrome (UWS) and minimally conscious state (MCS). Furthermore, in the local and global auditory stimulation paradigms, UWS and MCS patients respond differently to short-term and long-term auditory irregularities, exhibiting distinct HEP responses, which helps differentiate between UWS and MCS patients (Candia-Rivera et al., 2023; Raimondo et al., 2017).

### Other disorders

HEP has also been linked to pain perception, as afferent signals from the heart can modulate pain processing (Shao et al., 2011). In cold pain conditions, HEP in frontal and central regions shows an inhibitory state, with negative correlations between HEP amplitude and subjective pain ratings (Shao et al., 2011). Chronic pain patients tend to show reduced interoceptive sensitivity and lower HEP amplitudes (Solcà et al., 2020).

Short-term fasting and caloric intake impact cardiac autonomic function and the neural correlates of cardiac interoception, but the results are inconsistent (Flasbeck et al., 2021; Mai-Lippold et al., 2020). For example, one study found lower HEP amplitudes in right central and parietal electrodes after 16 hours of fasting, with an increase following nutritional intake (Flasbeck et al., 2021), while other studies reported opposite findings (Mai-Lippold et al., 2020; Schulz et al., 2015a). Moreover, clinical studies have identified marked interoceptive and emotional processing impairments in patients with binge eating disorder (Ortmann et al., 2025).

Patients with high-symptom medically unexplained symptoms (MUS) (Schulz et al., 2020) exhibit greater bodily signal sensitivity and attentional allocation to cardiac cues compared to low-symptom patients, despite no differences in IAc between groups.

SARS-CoV-2 infection (Kamp et al., 2023) was found to be associated with altered cardiac interoceptive processing and significantly reduce their attention and concentration performance.

## Discussion and conclusion

Experimental designs are important for analyzing the directionality of the BHI in HEP studies. In this review, we categorize these experimental designs into the following three types: (1) Bottom-up sensory input: studies investigated how interoceptive signals and external stimuli are passively transmitted to the brain and consequently affect HEP signals. These paradigms did not involve higher cognitive processes and only included sample tasks such as resting state, sleep, and passive visual, auditory, or other external sensory stimulation tasks. (2) Top-down predictive perception: studies explored how the brain predicts cardiac activity and external stimuli based on expectations, anticipations, and prior experiences. Commonly used experimental paradigms in such studies included mismatch and omission in regular sequences and repetition suppression paradigms. (3) Top-down regulation: studies involved the brain’s active regulation of interoceptive signals and external stimuli, such as observing the impact of cognitive or emotion-regulation tasks on HEP. Applying this taxonomy to the clinical literature included in our review shows a clear bias in current practice: roughly 63% of clinical HEP studies have employed bottom-up paradigms, ~35% have used regulation paradigms, whereas predictive perception paradigms remain markedly underrepresented (~2%).

Clinically, studies employing bottom-up sensory input and top-down regulation paradigms consistently demonstrate that HEP alterations are multidimensional and heterogeneous characteristics. Changes are not limited to increases or decreases in amplitude but also include latency shifts and spatial redistribution of cortical activity. Among these features, amplitude modulation is most frequently reported. For example, increased HEP amplitude is often observed in anxiety and trauma-related disorders, potentially reflecting heightened attention to or enhanced processing of internal bodily signals. In contrast, reduced amplitude is more commonly reported in cardiovascular diseases, certain depressive states, and chronic neurological conditions, possibly indicating attenuated cardiac afferent input or impaired cortical integration.

From a neuroanatomical perspective, HEP abnormalities primarily involve the insula, anterior cingulate cortex, central regions, and prefrontal areas. The right anterior insula, a key hub for interoceptive processing, shows consistent alterations across psychiatric and cardiovascular conditions. The anterior cingulate cortex, implicated in emotion–cognition integration, may reflect dysregulation in the integration of affective and bodily signals. Alterations in prefrontal control regions further underscore the role of higher-order regulatory mechanisms in interoceptive dysfunction. In cardiovascular disorders, HEP changes are more often characterized by attenuated responses in insular and sensory-related regions, whereas anxiety and trauma-related disorders more commonly exhibit enhanced or hyperreactive prefrontal–insular network activity, suggesting amplification of interoceptive signals by predictive and attentional mechanisms.

Despite substantial progress, clinical HEP research employing top-down predictive-perception paradigms remains scarce. The limited number of studies that do apply these designs have, for example, examined the relationship between negative emotional bias and interoceptive dysfunction in major depressive disorder (Zhou et al., 2022) and have used HEP to reveal differential processing of deviant auditory stimuli in patients with disorders of consciousness (Candia-Rivera et al., 2023) and in healthy individuals during sleep (Pelentritou et al., 2024). This relative paucity of predictive-paradigm work, however, constrains our ability to fully characterize disrupted predictive mechanisms in psychiatric and somatic disorders – many of which are marked by maladaptive expectation formation and misinterpretation of interoceptive signals.

## Supporting information

10.1017/S003329172610453X.sm001 Wang et al. supplementary material Wang et al. supplementary material

## Supplementary material

The supplementary material for this article can be found at http://doi.org/10.1017/S003329172610453X.

## Table 1. Long description

The table has three columns labeled Flow type, Task, and Task description. From top to bottom, the first section is Bottom-up sensory input, spanning three rows. Its tasks are Resting stats, Sleep processes, and Passive sensory tasks. Resting stats involve participants seated in a relaxed state without external stimuli. Sleep processes monitor physiological changes during sleep stages. Passive sensory tasks expose participants to sensory stimuli without requiring responses. The next section is Top-down predictive perception, spanning three rows. Tasks are Omission or mismatch task, Local–global paradigm, and Repetition suppression paradigm. Omission or mismatch tasks introduce omissions or mismatches in regular sequences. The Local–global paradigm presents local or global rule violations in sequences to study neural responses. The Repetition suppression paradigm presents repeated stimuli to investigate reduced neural activity. The final section is Top-down regulation, spanning five rows. Tasks are Heartbeat Counting Task, Heartbeat Attention Task, Heartbeat Tapping Task, Heartbeat Synchrony Task, and Other cognitive processing tasks. The Heartbeat Counting Task has participants silently count heartbeats. The Heartbeat Attention Task requires focus on heartbeats or external stimuli as cued. The Heartbeat Tapping Task involves tapping in sync with perceived heartbeats. The Heartbeat Synchrony Task distinguishes between synchronous or asynchronous stimuli with heartbeats. Other cognitive processing tasks involve attention, memory, or emotional evaluation with corresponding responses.

Navigate back to Table 1..

## Figure 1. Long description

Panel A, top-left, shows a seated figure with a thought bubble and heartbeat waveform labeled 1 to 6, representing silent heartbeat counting. Panel B, top-right, displays a heart icon and a head with a target, connected by dashed arrows to icons for external stimulation (ear, eye, nose, hand), indicating attention switching between heartbeat and external cues. Panel C, bottom-left, repeats the seated figure and waveform, with finger-tapping icons above each heartbeat peak, illustrating tapping in sync with perceived heartbeats. Panel D, bottom-right, presents the seated figure and waveform, with speaker icons above each peak. The synchronous section aligns speakers with heartbeat spikes; the asynchronous section offsets speakers from spikes, depicting discrimination between synchronous and asynchronous stimuli.

Navigate back to Figure 1..

## Figure 2. Long description

Panel A at the top-left shows three blocks labeled Baseline, Synchronous, and Asynchronous, each with timelines. Baseline has a 10-minute ECG trace with no sound stimulation. Synchronous and Asynchronous blocks (5–8 minutes each) show ECG traces with sound pulses aligned to R peaks; red-labeled ‘Omission’ marks missing sounds. Synchronous aligns sounds to heartbeats, Asynchronous uses baseline intervals. Panel B at bottom-left diagrams three trial types: (a) Repetition Trials with two face images separated by fixation crosses, labeled Stim 1 and Stim 2, with ISI and ITI intervals; (b) Alternation Trials with alternating faces; (c) Target (Catch) Trials with a button press prompt. All timings are in milliseconds. Panel C at right shows two blocks (X and Y) with sequences of green and red bars representing tones A/B and B/A over time. Flow arrows lead to scalp maps labeled Global standard Local standard (74 percent), Global deviant Local deviant (13 percent), and Omission (13 percent), with omission events highlighted in blue boxes. The lower half repeats with swapped deviant/standard assignments.

Navigate back to Figure 2..

## Figure 3. Long description

At the top, a yellow box states ‘Identification of studies via databases and registers.’ Below, the leftmost column begins with ‘526 Records identified from Databases (n=3): Web of science (n=208), Scopus (n=163), Pubmed (n=155).’ An arrow leads right to ‘Records removed before screening: Duplicate records removed (n=301).’ Downward, ‘Title and Abstract assessed for eligibility (n=225)' connects right to ‘Title and Abstract Records excluded (n=138): Not clinically relevant study, Not using H E P, System analysis and mate analysis, No peer review.’ Below, ‘Full-texts assessed for eligibility (n=87)' connects right to ‘Full-texts Records excluded (n=7): Not using H E P (n=3), Healthy Populations Using Cognitive and Clinical Scales (n=3), No results (n=1).’ At the bottom left, ‘Studies included in review (n=80): Regulatory strategies (n=16), Psychiatric disorders (n=15), Sleep-related (n=12), Cardiac and cardiovascular disorders (n=8), Age-related differences (n=6), Neurological disorders (n=10), Consciousness disorders (n=5), Other topics (n=8).’ Blue vertical labels on the left mark ‘Identification,’ ‘Screening,’ and ‘Included.’

Navigate back to Figure 3..
