# Supplementary material for: Heartbeat-evoked potentials reveal interoceptive dysfunction in clinical disorders: Experimental frameworks and promising applications
Source: Psychol Med. 2026 Jul 13;56:e216. doi: 10.1017/S003329172610453X (PMC13370194; doi:10.1017/S003329172610453X)
Supplement: Wang et al. supplementary material [file S003329172610453Xsup001.docx]

**Table 1.** Classification of the Included Studies According to Experimental Paradigm Types

| Clinical Application Types | Flow Types | References |
| --- | --- | --- |
| 1. Regulatory strategies | Bottom-up sensory input | (Flasbeck *et al.*, 2025, Gao *et al.*, 2023, Kang *et al.*, 2022, Majeed *et al.*, 2022, Schmitz *et al.*, 2020, Schulz *et al.*, 2013, Strohman *et al.*, 2024, Verdonk *et al.*, 2024, Wang *et al.*, 2024, Xu *et al.*, 2025, Yang *et al.*, 2025, Yoris *et al.*, 2024, Zhou *et al.*, 2024, Zwienenberg *et al.*, 2023) |
|  | Top-down regulation | (Kang *et al.*, 2022, Pollatos *et al.*, 2016, Richter *et al.*, 2021, Wang *et al.*, 2024, Zhou *et al.*, 2024) |
|  | Top-down predictive perception | / |
| 1. Psychiatric disorders | Bottom-up sensory input | (Cambi *et al.*, 2024, Flasbeck *et al.*, 2020, Koreki *et al.*, 2024, Müller *et al.*, 2015, Pang *et al.*, 2019, Schmitz *et al.*, 2021, Schulz *et al.*, 2015b, Schulz *et al.*, 2016) |
|  | Top-down regulation | (Herrera *et al.*, 2025, Judah *et al.*, 2018, Lutz *et al.*, 2019, Schulz *et al.*, 2022, Schulz *et al.*, 2015b, Schulz *et al.*, 2016, Terhaar *et al.*, 2012, Yoris *et al.*, 2017, Zhou *et al.*, 2022) |
|  | Top-down predictive perception | (Zhou *et al.*, 2022) |
| 1. Cardiac and cardiovascular disorders | Bottom-up sensory input | (Candia-Rivera and Machado, 2023b, Couto *et al.*, 2014, Kumral *et al.*, 2022, Limonova *et al.*, 2024, Schulz *et al.*, 2018) |
|  | Top-down regulation | (Couto *et al.*, 2014, Legaz *et al.*, 2022, Limonova *et al.*, 2024, Salamone *et al.*, 2020, Yoris *et al.*, 2018) |
|  | Top-down predictive perception | / |
| 1. Sleep-related | Bottom-up sensory input | (Abolfathi and Mohebbi, 2024, Baumert *et al.*, 2015, Billeci *et al.*, 2021, Bogdány *et al.*, 2022, Immanuel *et al.*, 2014, Lechinger *et al.*, 2015, Liu *et al.*, 2022, Perogamvros *et al.*, 2019, Seddighi and Mohebbi, 2024, Simor *et al.*, 2021, Simor *et al.*, 2025, Wei *et al.*, 2016) |
|  | Top-down regulation | / |
|  | Top-down predictive perception | / |
| 1. Neurological disorders | Bottom-up sensory input | (Birba *et al.*, 2022, Elkommos *et al.*, 2023, Flasbeck *et al.*, 2024, Melo *et al.*, 2022, Stoupi *et al.*, 2024, Wang *et al.*, 2025) |
|  | Top-down regulation | (Babo-Rebelo *et al.*, 2016, Birba *et al.*, 2022, Leopold and Schandry, 2001, Rapp *et al.*, 2023, Salamone *et al.*, 2018) |
|  | Top-down predictive perception | / |
| 1. Age-related differences | Bottom-up sensory input | (Kamp *et al.*, 2021, Mai *et al.*, 2018, Maister *et al.*, 2017, Weijs *et al.*, 2023) |
|  | Top-down regulation | (Aprile *et al.*, 2025, López Pérez *et al.*, 2023, Mai *et al.*, 2018) |
|  | Top-down predictive perception | / |
| 1. Consciousness disorder | Bottom-up sensory input | (Candia-Rivera *et al.*, 2021, Candia-Rivera and Machado, 2023a, Liuzzi *et al.*, 2024) |
|  | Top-down regulation | (Fló *et al.*, 2024) |
|  | Top-down predictive perception | (Candia-Rivera *et al.*, 2023) |
| 1. Other disorders | Bottom-up sensory input | (Flasbeck *et al.*, 2021, Mai-Lippold *et al.*, 2020, Ortmann *et al.*, 2025, Schulz *et al.*, 2015a, Schulz *et al.*, 2020, Shao *et al.*, 2011, Solcà *et al.*, 2020) |
|  | Top-down regulation | (Kamp *et al.*, 2023, Ortmann *et al.*, 2025, Schulz *et al.*, 2020, Solcà *et al.*, 2020) |
|  | Top-down predictive perception | / |

Note: Some studies involve experiments on various types of information flows

**Table 2.** The number of studies included in each experimental paradigm category and their commonly used task types.

| Flow type | Number of tasks | Commonly used task types |
| --- | --- | --- |
| Bottom-up sensory input | 58 | Resting stats, Sleep Processes, Passive sensory tasks (Such as external electrical stimulation or audio-visual stimulation) |
| Top-down regulation | 32 | Heartbeat Counting Task, Heartbeat Synchrony Task, Other cognitive processing tasks |
| Top-down predictive perception | 2 | Local-global paradigm, mismatch task |

Note: Some studies involve experiments on various types of information flows

**Abolfathi, Y. & Mohebbi, M.** (2024). Heightened Heartbeat Evoked Potential in Obstructive Sleep Apnea Disorder during Sleep. *IEEE Access* **12**, 189153-189162.

**Aprile, F., Simões, M., Henriques, J., Carvalho, P., Castelo-Branco, M., Sel, A. & Ribeiro, M. J.** (2025). The Heartbeat-Evoked Potential in Young and Older Adults During Attention Orienting. *Psychophysiology* **62**, e70057.

**Babo-Rebelo, M., Wolpert, N., Adam, C., Hasboun, D. & Tallon-Baudry, C.** (2016). Is the cardiac monitoring function related to the self in both the default network and right anterior insula? *Philosophical Transactions of the Royal Society B: Biological Sciences* **371**.

**Baumert, M., Pamula, Y., Kohler, M., Martin, J., Kennedy, D., Nalivaiko, E. & Immanuel, S. A.** (2015). Effect of respiration on heartbeat-evoked potentials during sleep in children with sleep-disordered breathing. *Sleep Medicine* **16**, 665-667.

**Billeci, L., Faraguna, U., Santarcangelo, E. L., d'Ascanio, P., Varanini, M. & Sebastiani, L.** (2021). Heartbeat-Evoked Cortical Potential during Sleep and Interoceptive Sensitivity: A Matter of Hypnotizability. *Brain Sciences* **11**.

**Birba, A., Santamaria-Garcia, H., Prado, P., Cruzat, J., Sainz Ballesteros, A., Legaz, A., Fittipaldi, S., Duran-Aniotz, C., Slachevsky, A., Santibanez, R., Sigman, M., Garcia, A. M., Whelan, R., Moguilner, S. & Ibanez, A.** (2022). Allostatic-Interoceptive Overload in Frontotemporal Dementia. *Biological Psychiatry* **92**, 54-67.

**Bogdány, T., Perakakis, P., Bódizs, R. & Simor, P.** (2022). The heartbeat evoked potential is a questionable biomarker in nightmare disorder: A replication study. *NeuroImage: Clinical* **33**.

**Cambi, S., Solcà, M., Micali, N. & Berchio, C.** (2024). Cardiac interoception in Anorexia Nervosa: A resting-state heartbeat-evoked potential study. *European Eating Disorders Review* **32**, 417-430.

**Candia-Rivera, D., Annen, J., Gosseries, O., Martial, C., Thibaut, A., Laureys, S. & Tallon-Baudry, C.** (2021). Neural responses to heartbeats detect residual signs of consciousness during resting state in postcomatose patients. *Journal of Neuroscience* **41**, 5251-5262.

**Candia-Rivera, D. & Machado, C.** (2023a). Multidimensional assessment of heartbeat-evoked responses in disorders of consciousness. *European Journal of Neuroscience* **58**, 3098-3110.

**Candia-Rivera, D. & Machado, C.** (2023b). Reduced Heartbeat-Evoked Responses in a Near-Death Case Report. *Journal of Clinical Neurology (Korea)* **19**, 581-588.

**Candia-Rivera, D., Raimondo, F., Pérez, P., Naccache, L., Tallon-Baudry, C. & Sitt, J. D.** (2023). Conscious processing of global and local auditory irregularities causes differentiated heartbeat-evoked responses. *eLife* **12**.

**Couto, B., Salles, A., Sedeno, L., Peradejordi, M., Barttfeld, P., Canales-Johnson, A., Dos Santos, Y. V., Huepe, D., Bekinschtein, T., Sigman, M., Favaloro, R., Manes, F. & Ibanez, A.** (2014). The man who feels two hearts: the different pathways of interoception. *Social Cognitive and Affective Neuroscience* **9**, 1253-1260.

**Elkommos, S., Martin-Lopez, D., Koreki, A., Jolliffe, C., Kandasamy, R., Mula, M., Critchley, H. D., Edwards, M. J., Garfinkel, S., Richardson, M. P. & Yogarajah, M.** (2023). Changes in the heartbeat-evoked potential are associated with functional seizures. *Journal of Neurology, Neurosurgery and Psychiatry* **94**, 769-775.

**Flasbeck, V., Bamberg, C. & Brüne, M.** (2021). Short-Term Fasting and Ingestion of Caloric Drinks Affect Heartbeat-Evoked Potentials and Autonomic Nervous System Activity in Males. *Frontiers in Neuroscience* **15**.

**Flasbeck, V., Jungilligens, J., Lemke, I., Beckers, J., Öztürk, H., Wellmer, J., Seliger, C., Juckel, G. & Popkirov, S.** (2024). Heartbeat evoked potentials and autonomic arousal during dissociative seizures: insights from electrophysiology and neuroimaging. *BMJ Neurol Open* **6**, e000665.

**Flasbeck, V., Popkirov, S., Ebert, A. & Brüne, M.** (2020). Altered interoception in patients with borderline personality disorder: a study using heartbeat-evoked potentials. *Borderline Personality Disorder and Emotion Dysregulation* **7**, 24.

**Flasbeck, V., Schedlowski, M., Brüne, M. & Engler, H.** (2025). Impact of experimental inflammation on the neuronal processing of cardiac interoceptive signals and heart rate variability in humans. *NeuroImage* **314**, 121257.

**Fló, E., Belloli, L., Cabana, Á., Ruyant-Belabbas, A., Jodaitis, L., Valente, M., Rohaut, B., Naccache, L., Rosanova, M., Comanducci, A., Andrillon, T. & Sitt, J.** (2024). Predicting attentional focus: Heartbeat-evoked responses and brain dynamics during interoceptive and exteroceptive processing. *PNAS Nexus* **3**, pgae531.

**Gao, J., Sun, R., Leung, H. K., Roberts, A., Wu, B. W. Y., Tsang, E. W., Tang, A. C. W. & Sik, H. H.** (2023). Increased neurocardiological interplay after mindfulness meditation: a brain oscillation-based approach. *Frontiers in Human Neuroscience* **17**.

**Herrera, E., Gutierrez-Sterling, D., Barrera-Ocampo, A., Jaramillo, J. O., Santamaría-García, H. & Birba, A.** (2025). Impaired interoception in Colombian victims of armed conflict with PTSD: a preliminary HEP study. *Frontiers in Psychology* **16**.

**Immanuel, S. A., Pamula, Y., Kohler, M., Martin, J., Kennedy, D., Nalivaiko, E., Saint, D. A. & Baumert, M.** (2014). Heartbeat evoked potentials during sleep and daytime behavior in children with sleep-disordered breathing. *American Journal of Respiratory and Critical Care Medicine* **190**, 1149-1157.

**Judah, M. R., Shurkova, E. Y., Hager, N. M., White, E. J., Taylor, D. L. & Grant, D. M.** (2018). The relationship between social anxiety and heartbeat evoked potential amplitude. *Biological Psychology* **139**, 1-7.

**Kamp, S. M., Buntić, N., Amtmann, J., Scharpf, A., Schönen, A., Wagner, L. & Schulz, A.** (2023). Reduced concentration performance and heartbeat-evoked potential in individuals with a history of a SARS-CoV-2 infection. *Neuroscience Letters* **814**.

**Kamp, S. M., Schulz, A., Forester, G. & Domes, G.** (2021). Older adults show a higher heartbeat-evoked potential than young adults and a negative association with everyday metacognition. *Brain Research* **1752**, 147238.

**Kang, S. S., Sponheim, S. R. & Lim, K. O.** (2022). Interoception Underlies Therapeutic Effects of Mindfulness Meditation for Posttraumatic Stress Disorder: A Randomized Clinical Trial. *Biological Psychiatry: Cognitive Neuroscience and Neuroimaging* **7**, 793-804.

**Koreki, A., Ogyu, K., Miyazaki, T., Takenouchi, K., Matsushita, K., Honda, S., Koizumi, T., Onaya, M., Uchida, H., Mimura, M., Nakajima, S. & Noda, Y.** (2024). Aberrant heartbeat-evoked potential in schizophrenia. *Progress in Neuro-Psychopharmacology and Biological Psychiatry* **132**.

**Kumral, D., Al, E., Cesnaite, E., Kornej, J., Sander, C., Hensch, T., Zeynalova, S., Tautenhahn, S., Hagendorf, A., Laufs, U., Wachter, R., Nikulin, V. & Villringer, A.** (2022). Attenuation of the Heartbeat-Evoked Potential in Patients With Atrial Fibrillation. *JACC: Clinical Electrophysiology* **8**, 1219-1230.

**Lechinger, J., Heib, D. P. J., Gruber, W., Schabus, M. & Klimesch, W.** (2015). Heartbeat-related EEG amplitude and phase modulations from wakefulness to deep sleep: Interactions with sleep spindles and slow oscillations. *Psychophysiology* **52**, 1441-1450.

**Legaz, A., Yoris, A., Sedeno, L., Abrevaya, S., Martorell, M., Alifano, F., Garcia, A. M. & Ibanez, A.** (2022). Heart-brain interactions during social and cognitive stress in hypertensive disease: A multidimensional approach. *European Journal of Neuroscience* **55**, 2836-2850.

**Leopold, C. & Schandry, R.** (2001). The heartbeat-evoked brain potential in patients suffering from diabetic neuropathy and in healthy control persons. *Clinical Neurophysiology* **112**, 674-682.

**Limonova, A. S., Minenko, I. A., Sukmanova, A. A., Kutsenko, V. A., Kulikova, S. P., Nazarova, M. A., Davtyan, K. V., Drapkina, O. M. & Ershova, A. I.** (2024). Exploring the Link Between Interoception and Symptom Severity in Premature Ventricular Contractions. *Journal of Clinical Medicine* **13**.

**Liu, H., Yu, X., Wang, G., Han, Y. & Wang, W.** (2022). Effects of 24-h acute total sleep deprivation on physiological coupling in healthy young adults. *Frontiers in Neuroscience* **16**.

**Liuzzi, P., Cassioli, T., Secci, S., Hakiki, B., Scarpino, M., Burali, R., di Palma, A., Toci, T., Grippo, A., Cecchi, F., Frosini, A. & Mannini, A.** (2024). A neurophysiological profiling of the heartbeat-evoked potential in severe acquired brain injuries: A focus on unconsciousness. *European Journal of Neuroscience* **60**, 4201-4216.

**López Pérez, D., Bokde, A. L. W. & Kerskens, C. M.** (2023). Complexity analysis of heartbeat-related signals in brain MRI time series as a potential biomarker for ageing and cognitive performance. *European Physical Journal: Special Topics* **232**, 123-133.

**Lutz, A. P. C., Schulz, A., Voderholzer, U., Koch, S., van Dyck, Z. & Vögele, C.** (2019). Enhanced cortical processing of cardio-afferent signals in anorexia nervosa. *Clinical Neurophysiology* **130**, 1620-1627.

**Mai-Lippold, S. A., Dettlinger, C. M., Khalsa, S. S. & Pollatos, O.** (2020). A Pilot Study on the Effect of an Energy Drink on Interoception in High vs. Low Anxiety Sensitivity Individuals. *European Journal of Health Psychology* **27**, 171-187.

**Mai, S., Wong, C. K., Georgiou, E. & Pollatos, O.** (2018). Interoception is associated with heartbeat-evoked brain potentials (HEPs) in adolescents. *Biological Psychology* **137**, 24-33.

**Maister, L., Tang, T. & Tsakiris, M.** (2017). Neurobehavioral evidence of interoceptive sensitivity in early infancy. *Elife* **6**.

**Majeed, U., Aftab, M. F., Baloch, D. M., Ahmed, S., Yusuf, I. M., Hasan, M. A. & Qureshi, M. S.** (2022). Modulation of Heart and Brain Function by Surah Al-Rehman Recitation Among Distressed Diabetic Patients in Pakistan. *Journal of Religion and Health* **61**, 3852-3865.

**Melo, E., Fiel, J., Milhomens, R., Ribeiro, T., Navegantes, R., Gomes, F., Gomes, B. D. & Pereira, A.** (2022). Dynamic coupling between the central and autonomic cardiac nervous systems in patients with refractory epilepsy: A pilot study. *Frontiers in Neurology* **13**.

**Müller, L. E., Schulz, A., Andermann, M., Gäbel, A., Gescher, D. M., Spohn, A., Herpertz, S. C. & Bertsch, K.** (2015). Cortical representation of afferent bodily signals in borderline personality disorder: Neural correlates and relationship to emotional dysregulation. *JAMA Psychiatry* **72**, 1077-1086.

**Ortmann, J., Schulz, A., Lutz, A. P. C., van Dyck, Z. & Vögele, C.** (2025). Cardiac interoceptive processing and emotional experience in binge eating behavior: Neural evidence of disengagement from bodily sensations. *Appetite* **208**, 107948.

**Pang, J., Tang, X., Li, H., Hu, Q., Cui, H., Zhang, L., Li, W., Zhu, Z., Wang, J. & Li, C.** (2019). Altered Interoceptive Processing in Generalized Anxiety Disorder-A Heartbeat-Evoked Potential Research. *Front Psychiatry* **10**, 616.

**Perogamvros, L., Park, H. D., Bayer, L., Perrault, A. A., Blanke, O. & Schwartz, S.** (2019). Increased heartbeat-evoked potential during REM sleep in nightmare disorder. *NeuroImage: Clinical* **22**.

**Pollatos, O., Herbert, B. M., Mai, S. & Kammer, T.** (2016). Changes in interoceptive processes following brain stimulation. *Philos Trans R Soc Lond B Biol Sci* **371**.

**Rapp, L., Mai-Lippold, S. A., Georgiou, E. & Pollatos, O.** (2023). Elevated EEG heartbeat-evoked potentials in adolescents with more ADHD symptoms. *Biological Psychology* **184**.

**Richter, F., García, A. M., Rodriguez Arriagada, N., Yoris, A., Birba, A., Huepe, D., Zimmer, H., Ibáñez, A. & Sedeño, L.** (2021). Behavioral and neurophysiological signatures of interoceptive enhancements following vagus nerve stimulation. *Human Brain Mapping* **42**, 1227-1242.

**Salamone, P. C., Esteves, S., Sinay, V. J., García-Cordero, I., Abrevaya, S., Couto, B., Adolfi, F., Martorell, M., Petroni, A., Yoris, A., Torquati, K., Alifano, F., Legaz, A., Cassará, F. P., Bruno, D., Kemp, A. H., Herrera, E., García, A. M., Ibáñez, A. & Sedeño, L.** (2018). Altered neural signatures of interoception in multiple sclerosis. *Human Brain Mapping* **39**, 4743-4754.

**Salamone, P. C., Sedeño, L., Legaz, A., Bekinschtein, T., Martorell, M., Adolfi, F., Fraile-Vazquez, M., Rodríguez Arriagada, N., Favaloro, L., Peradejordi, M., Absi, D. O., García, A. M., Favaloro, R. & Ibáñez, A.** (2020). Dynamic neurocognitive changes in interoception after heart transplant. *Brain Communications* **2**.

**Schmitz, M., Müller, L. E., Schulz, A., Kleindienst, N., Herpertz, S. C. & Bertsch, K.** (2020). Heart and brain: Cortical representation of cardiac signals is disturbed in borderline personality disorder, but unaffected by oxytocin administration. *Journal of Affective Disorders* **264**, 24-28.

**Schmitz, M., Müller, L. E., Seitz, K. I., Schulz, A., Steinmann, S., Herpertz, S. C. & Bertsch, K.** (2021). Heartbeat evoked potentials in patients with post-traumatic stress disorder: an unaltered neurobiological regulation system? *European Journal of Psychotraumatology* **12**.

**Schulz, A., de Sa, D. S. F., Dierolf, A. M., Lutz, A., van Dyck, Z., Voegele, C. & Schaechinger, H.** (2015a). Short-term food deprivation increases amplitudes of heartbeat-evoked potentials. *Psychophysiology* **52**, 695-703.

**Schulz, A., Dierolf, A. M., Lutz, A. P. C., Voderholzer, U., Koch, S., Bach, M., Asenstorfer, C., Michaux, G., Mertens, V. C. & Vögele, C.** (2022). Higher cardiovascular activation, but normal heartbeat-evoked potentials and cardiac interoceptive accuracy in somatoform disorders and major depressive disorder. *Psychiatry Research Communications* **2**.

**Schulz, A., Koester, S., Beutel, M. E., Schaechinger, H., Voegele, C., Rost, S., Rauh, M. & Michal, M.** (2015b). Altered Patterns of Heartbeat-Evoked Potentials in Depersonalization/Derealization Disorder: Neurophysiological Evidence for Impaired Cortical Representation of Bodily Signals. *Psychosomatic Medicine* **77**, 506-516.

**Schulz, A., Mattheyc, J. H., Voegele, C., Schaan, V., Schaechinger, H., Adler, J., Beutel, M. E. & Michal, M.** (2016). Cardiac modulation of startle is altered in depersonalization-/derealization disorder: Evidence for impaired brainstem representation of baro-afferent neural traffic. *Psychiatry Research* **240**, 4-10.

**Schulz, A., Rost, S., Flasinski, T., Dierolf, A. M., Lutz, A. P. C., Münch, E. E., Mertens, V. C., Witthöft, M. & Vögele, C.** (2020). Distinctive body perception mechanisms in high versus low symptom reporters: A neurophysiological model for medically-unexplained symptoms. *Journal of Psychosomatic Research* **137**.

**Schulz, A., Stammet, P., Dierolf, A. M., Vögele, C., Beyenburg, S., Werer, C. & Devaux, Y.** (2018). Late heartbeat-evoked potentials are associated with survival after cardiac arrest. *Resuscitation* **126**, 7-13.

**Schulz, A., Strelzyk, F., Ferreira de Sá, D. S., Naumann, E., Vögele, C. & Schächinger, H.** (2013). Cortisol rapidly affects amplitudes of heartbeat-evoked brain potentials-Implications for the contribution of stress to an altered perception of physical sensations? *Psychoneuroendocrinology* **38**, 2686-2693.

**Seddighi, B. & Mohebbi, M.** (2024). Investigation of Heartbeat Evoked Potential (HEP) Response During Different Stages of Sleep in Sleep Disorders. *Frontiers in Biomedical Technologies* **11**, 302-314.

**Shao, S., Shen, K., Wilder-Smith, E. P. V. & Li, X.** (2011). Effect of pain perception on the heartbeat evoked potential. *Clinical Neurophysiology* **122**, 1838-1845.

**Simor, P., Bogdány, T., Bódizs, R. & Perakakis, P.** (2021). Cortical monitoring of cardiac activity during rapid eye movement sleep: The heartbeat evoked potential in phasic and tonic rapid-eye-movement microstates. *Sleep* **44**.

**Simor, P., Lilla, R. Z., Szalárdy, O., Jordán, Z., Halász, L., Erőss, L., Fabó, D. & Bódizs, R.** (2025). Heartbeat-related activity in the anterior thalamus differs between phasic and tonic REM sleep. *Journal of Physiology* **603**, 2839-2855.

**Solcà, M., Park, H. D., Bernasconi, F. & Blanke, O.** (2020). Behavioral and neurophysiological evidence for altered interoceptive bodily processing in chronic pain. *NeuroImage* **217**, 116902.

**Stoupi, N. A., Weijs, M. L., Imbach, L. & Lenggenhager, B.** (2024). Heartbeat-evoked potentials following voluntary hyperventilation in epilepsy patients: respiratory influences on cardiac interoception. *Frontiers in Neuroscience* **18**.

**Strohman, A., Isaac, G., Payne, B., Verdonk, C., Khalsa, S. S. & Legon, W.** (2024). Low-intensity focused ultrasound to the insula differentially modulates the heartbeat-evoked potential: A proof-of-concept study. *Clinical Neurophysiology* **167**, 267-281.

**Terhaar, J., Viola, F. C., Bär, K. J. & Debener, S.** (2012). Heartbeat evoked potentials mirror altered body perception in depressed patients. *Clinical Neurophysiology* **123**, 1950-1957.

**Verdonk, C., Teed, A. R., White, E. J., Ren, X., Stewart, J. L., Paulus, M. P. & Khalsa, S. S.** (2024). Heartbeat-evoked neural response abnormalities in generalized anxiety disorder during peripheral adrenergic stimulation. *Neuropsychopharmacology* **49**, 1246-1254.

**Wang, M. Y., Corcoran, A. W., McQueen, B., Freedman, G., Humble, G., Fitzgibbon, B. M., Fitzgerald, P. B. & Bailey, N. W.** (2024). Experienced Meditators Show Enhanced Interaction Between Brain and Heart Functioning. *Mindfulness* **15**, 3198-3216.

**Wang, X., Yang, H., Cheng, Y., Liu, S., Jin, G., Qiao, Z., Qi, L., Wang, S., Ge, J., Hu, D., Tang, H., Gao, R., Xu, C., Zhang, X., Wang, D., Xue, X., Dai, A., Zhao, W., Yu, T., Wang, Y., Si, B., Zhao, G. & Ren, L.** (2025). Mapping human brain topography to heart rhythms: an SEEG study. *Cardiovascular Research*.

**Wei, Y., Ramautar, J. R., Colombo, M. A., Stoffers, D., Gómez-Herrero, G., Van Der Meijden, W. P., Te Lindert, B. H. W., Van Der Werf, Y. D. & Van Someren, E. J. W.** (2016). I keep a close watch on this heart of mine: Increased interoception in Insomnia. *Sleep* **39**, 2113-2124.

**Weijs, M. L., Daum, M. M. & Lenggenhager, B.** (2023). Cardiac interoception in infants: Behavioral and neurophysiological measures in various emotional and self-related contexts. *Psychophysiology* **60**, e14386.

**Xu, E., Pitts, S., Dahill-Fuchel, J., Scherrer, S., Nauvel, T., Overton, J. G., Riva-Posse, P., Crowell, A., Figee, M., Alagapan, S., Rozell, C. J., Choi, K. S., Mayberg, H. S. & Waters, A. C.** (2025). Neural Interoceptive Processing Is Modulated by Deep Brain Stimulation to Subcallosal Cingulate Cortex for Treatment-Resistant Depression. *Biological Psychiatry: Cognitive Neuroscience and Neuroimaging* **10**, 495-503.

**Yang, H., Herbelin, B., Ngo, C., Vuarnesson, L. & Blanke, O.** (2025). Meditation in the third-person perspective modulates minimal self and heartbeat-evoked potentials. *Neuroimage* **314**.

**Yoris, A., Abrevaya, S., Esteves, S., Salamone, P., Lori, N., Martorell, M., Legaz, A., Alifano, F., Petroni, A., Sanchez, R., Sedeno, L., Garcia, A. M. & Ibanez, A.** (2018). Multilevel convergence of interoceptive impairments in hypertension: New evidence of disrupted body-brain interactions. *Human Brain Mapping* **39**, 1563-1581.

**Yoris, A., Garcia, A. M., Traiber, L., Santamaria-Garcia, H., Martorell, M., Alifano, F., Kichic, R., Moser, J. S., Cetkovich, M., Manes, F., Ibanez, A. & Sedeno, L.** (2017). The inner world of overactive monitoring: neural markers of interoception in obsessive-compulsive disorder. *Psychological Medicine* **47**, 1957-1970.

**Yoris, A. E., Cira, L. F., Luque-Casado, A., Salvotti, C., Tajadura-Jimenez, A., Avancini, C., Zarza-Rebollo, J. A., Sanabria, D. & Perakakis, P.** (2024). Delving into the relationship between regular physical exercise and cardiac interoception in two cross-sectional studies. *Neuropsychologia* **198**.

**Zhou, H., Zou, H., Dai, Z., Zhao, S., Hua, L., Xia, Y., Han, Y., Yan, R., Tang, H., Huang, Y., Du, Y., Wang, X., Yao, Z. & Lu, Q.** (2022). Interoception Dysfunction Contributes to the Negative Emotional Bias in Major Depressive Disorder. *Frontiers in Psychiatry* **13**, 874859.

**Zhou, M., Cheng, L., Zhou, Y., Zhu, S., Zhang, Y., Kendrick, K. M. & Yao, S.** (2024). Intranasal Oxytocin Improves Interoceptive Accuracy and Heartbeat-Evoked Potentials During a Cardiac Interoceptive Task. *Biological Psychiatry: Cognitive Neuroscience and Neuroimaging* **9**, 1019-1027.

**Zwienenberg, L., Van Dijk, H., Enriquez-Geppert, S., Van Der Vinne, N., Gevirtz, R., Gordon, E., Sack, A. T. & Arns, M.** (2023). Heartbeat-Evoked Potential in Major Depressive Disorder: A Biomarker for Differential Treatment Prediction between Venlafaxine and rTMS? *Neuropsychobiology* **82**, 158-167.
